# (*Z*)-1-(2,4-Difluoro­phen­yl)-3-(4-fluoro­phen­yl)-2-(1*H*-1,2,4-triazol-1-yl)prop-2-en-1-one

**DOI:** 10.1107/S1600536812012123

**Published:** 2012-03-28

**Authors:** Ben-Tao Yin, Jing-Song Lv, Yan Wang, Cheng-He Zhou

**Affiliations:** a Laboratory of Bioorganic & Medicinal Chemistry, School of Chemistry and Chemical Engineering, Southwest University, Chongqing 400715, People’s Republic of China

## Abstract

In the title mol­ecule, C_17_H_10_F_3_N_3_O, the C=C bond connecting the triazole ring and 4-fluoro­phenyl groups adopts a *Z* conformation. The triazole ring forms dihedral angles of 15.3 (1) and 63.5 (1)°, with the 2,4-difluoro-substituted and 4-fluoro-substituted benzene rings, respectively. The dihedral angle between the two benzene rings is 51.8 (1)°.

## Related literature

For the pharmacological activity of triazole derivatives, see: Wang & Zhou (2011 ▶); Zhou & Wang (2012 ▶). For the biological activity of chalcones, see: Jin *et al.* (2010 ▶). For related structures, see: Wang *et al.* (2009 ▶); Yan *et al.* (2009 ▶). For the synthesis, see: Yan *et al.* (2009 ▶).
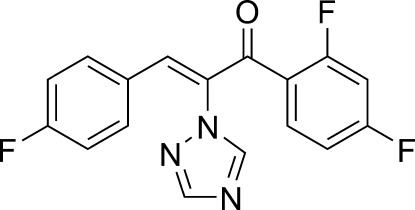


## Experimental

### 

#### Crystal data

C_17_H_10_F_3_N_3_O
*M* *_r_* = 329.28
Monoclinic, 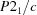

*a* = 11.735 (6) Å
*b* = 7.698 (4) Å
*c* = 17.065 (7) Å
β = 110.45 (3)°
*V* = 1444.4 (12) Å^3^
*Z* = 4
Mo *K*α radiation
μ = 0.12 mm^−1^
*T* = 293 K
0.19 × 0.17 × 0.16 mm

#### Data collection

Bruker APEXII CCD diffractometer
Absorption correction: multi-scan (*SADABS*; Bruker, 2009 ▶) *T*_min_ = 0.977, *T*_max_ = 0.980
7601 measured reflections
2840 independent reflections
2095 reflections with *I* > 2σ(*I*)
*R*_int_ = 0.027

#### Refinement

*R*[*F* ^2^ > 2σ(*F* ^2^)] = 0.042
*wR*(*F* ^2^) = 0.100
*S* = 1.01
2840 reflections
217 parameters
H-atom parameters constrained
Δρ_max_ = 0.15 e Å^−3^
Δρ_min_ = −0.21 e Å^−3^

### 

Data collection: *APEX2* (Bruker, 2009 ▶); cell refinement: *SAINT* (Bruker, 2009 ▶); data reduction: *SAINT*; program(s) used to solve structure: *SHELXS97* (Sheldrick, 2008 ▶); program(s) used to refine structure: *SHELXL97* (Sheldrick, 2008 ▶); molecular graphics: *SHELXTL* (Sheldrick, 2008 ▶); software used to prepare material for publication: *SHELXTL*.

## Supplementary Material

Crystal structure: contains datablock(s) global, I. DOI: 10.1107/S1600536812012123/lh5435sup1.cif

Structure factors: contains datablock(s) I. DOI: 10.1107/S1600536812012123/lh5435Isup2.hkl

Supplementary material file. DOI: 10.1107/S1600536812012123/lh5435Isup3.cml

Additional supplementary materials:  crystallographic information; 3D view; checkCIF report

## Figures and Tables

**Fig. 1.**
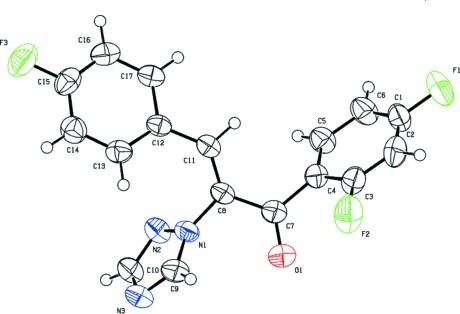
The molecular structure of (I), showing the displacement ellipsoids are drawn at the 50% probability level.

## supplementary crystallographic information

### Comment

Triazole compounds exhibit broad bioactive spectrum and the developments of new triazole derivatives as potential bioactive agents have become an active topic in medicinal chemistry (Wang & Zhou, 2011; Zhou & Wang, 2012). Chalcones have been paid increasingly special attention for their diverse biological activities such as antimicrobial, anticancer, antiviral and anti-inflammatory ones and so on (Jin *et al.*, 2010). Our interest is to develop novel triazole-derived chalcone compounds as medicinal agents. We have already synthesized and reported related structures of triazolylchalcones (Wang *et al.*, 2009; Yan *et al.*, 2009). Herein, we report the crystal structure of the title compound (I).

The molecular structure of (I) is shown in Fig. 1. The C═C bond connecting the triazole ring and 4-fluorophenyl groups adopts a *Z* geometry. The atoms in the region of the C═C bond have an essentially planar arrangement i.e. the r.m.s. deviation the atoms C7/C8/C11/C12/N1 is 0.0480 Å. The torsion angles of C12–C11═C8–C7 and C12–C11═C8–N1 are -169.35 (16)° and 7.1 (3)°. The triazole ring forms dihedral angles of 15.3 (1)° and 63.5 (1)°, with the 2,4-difluoro substituted (C1-C6) and 4-fluoro substituted (C12-C17) benzene rings, respectively. The dihedral angles between the two benzene rings is 51.8 (1)°.

### Experimental

Compound (I) was prepared according to the procedure of Yan *et al.* (2009). A mixture of 1-(2,4-difluorophenyl)-2-(1H-1,2,4-triazol-1-yl) ethanone (3.07 g, 13.8 mmol) and 4-fluorobenzaldehyde (1.89 g, 15.3 mmol) in toluene (30 mL) using glacial acetic acid (0.08 mL, 1.4 mmol) as catalyst was refluxed. After the reaction was complete (monitored by TLC, petroleum ether/ethyl acetate, 10/1, V/V), the solvent was removed. The residue was dissolved in dichloromethane (30 mL) and washed with water (3x30 mL). The resulting phase was dried over anhydrous sodium sulfate, concentrated under reduced pressure and then purified by silica gel column chromatography eluting with petroleum ether/ethyl acetate (10/1-2/1, V/V) to give the title compound (I) (2.578 g) as solid. Crystals suitable for X-ray analysis were grown from a mixed solution of (I) in ethyl acetate and petroleum ether by slow evaporation at room temperature.

### Refinement

H atoms were placed in calculated positions with C—H = 0.93 Å. The *U*_iso_(H) value was set equal to 1.2U_eq_(C).

### Crystal data

**Table:**

C_17_H_10_F_3_N_3_O	*F*(000) = 672
*M**_r_* = 329.28	*D*_x_ = 1.514 Mg m^−^^3^
Monoclinic, *P*2_1_/*c*	Mo *K*α radiation, λ = 0.71073 Å
Hall symbol: -P 2ybc	Cell parameters from 2158 reflections
*a* = 11.735 (6) Å	θ = 2.9–23.2°
*b* = 7.698 (4) Å	µ = 0.12 mm^−^^1^
*c* = 17.065 (7) Å	*T* = 293 K
β = 110.45 (3)°	Block, colourless
*V* = 1444.4 (12) Å^3^	0.19 × 0.17 × 0.16 mm
*Z* = 4	

### Data collection

**Table:**

Bruker APEXII CCD diffractometer	2840 independent reflections
Radiation source: fine-focus sealed tube	2095 reflections with *I* > 2σ(*I*)
Graphite monochromator	*R*_int_ = 0.027
φ and ω scans	θ_max_ = 26.0°, θ_min_ = 2.6°
Absorption correction: multi-scan (*SADABS*; Bruker, 2009)	*h* = −14→14
*T*_min_ = 0.977, *T*_max_ = 0.980	*k* = −9→9
7601 measured reflections	*l* = −21→16

### Refinement

**Table:**

Refinement on *F*^2^	Primary atom site location: structure-invariant direct methods
Least-squares matrix: full	Secondary atom site location: difference Fourier map
*R*[*F*^2^ > 2σ(*F*^2^)] = 0.042	Hydrogen site location: inferred from neighbouring sites
*wR*(*F*^2^) = 0.100	H-atom parameters constrained
*S* = 1.01	*w* = 1/[σ^2^(*F*_o_^2^) + (0.0398*P*)^2^ + 0.3484*P*] where *P* = (*F*_o_^2^ + 2*F*_c_^2^)/3
2840 reflections	(Δ/σ)_max_ = 0.001
217 parameters	Δρ_max_ = 0.15 e Å^−^^3^
0 restraints	Δρ_min_ = −0.21 e Å^−^^3^

### Special details

**Table:**

Geometry. All e.s.d.'s (except the e.s.d. in the dihedral angle between two l.s. planes) are estimated using the full covariance matrix. The cell e.s.d.'s are taken into account individually in the estimation of e.s.d.'s in distances, angles and torsion angles; correlations between e.s.d.'s in cell parameters are only used when they are defined by crystal symmetry. An approximate (isotropic) treatment of cell e.s.d.'s is used for estimating e.s.d.'s involving l.s. planes.
Refinement. Refinement of *F*^2^ against ALL reflections. The weighted *R*-factor *wR* and goodness of fit *S* are based on *F*^2^, conventional *R*-factors *R* are based on *F*, with *F* set to zero for negative *F*^2^. The threshold expression of *F*^2^ > σ(*F*^2^) is used only for calculating *R*-factors(gt) *etc*. and is not relevant to the choice of reflections for refinement. *R*-factors based on *F*^2^ are statistically about twice as large as those based on *F*, and *R*- factors based on ALL data will be even larger.

### Fractional atomic coordinates and isotropic or equivalent isotropic displacement parameters (Å^2^)

**Table:**

	*x*	*y*	*z*	*U*_iso_*/*U*_eq_	
F1	−0.10204 (11)	0.63601 (19)	0.88238 (9)	0.0882 (4)	
F2	0.09918 (11)	0.6680 (2)	0.69415 (7)	0.0836 (4)	
F3	0.87808 (11)	1.03042 (17)	1.09542 (8)	0.0816 (4)	
O1	0.29519 (13)	0.4323 (2)	0.74119 (9)	0.0862 (5)	
N1	0.52550 (12)	0.49839 (18)	0.83262 (8)	0.0416 (3)	
N2	0.61373 (13)	0.40221 (19)	0.88775 (9)	0.0498 (4)	
N3	0.64451 (15)	0.4248 (2)	0.76604 (10)	0.0619 (5)	
C1	−0.00130 (16)	0.6098 (3)	0.86429 (13)	0.0551 (5)	
C2	−0.00402 (16)	0.6532 (3)	0.78670 (12)	0.0579 (5)	
H2A	−0.0732	0.7000	0.7469	0.070*	
C3	0.09943 (16)	0.6248 (2)	0.77016 (10)	0.0508 (4)	
C4	0.20312 (15)	0.5587 (2)	0.82741 (10)	0.0432 (4)	
C5	0.20019 (16)	0.5199 (2)	0.90521 (11)	0.0493 (4)	
H5A	0.2696	0.4763	0.9460	0.059*	
C6	0.09753 (18)	0.5442 (3)	0.92381 (12)	0.0576 (5)	
H6A	0.0958	0.5161	0.9764	0.069*	
C7	0.30928 (16)	0.5142 (2)	0.80365 (11)	0.0487 (4)	
C8	0.43130 (14)	0.5720 (2)	0.85556 (9)	0.0392 (4)	
C9	0.54611 (17)	0.5086 (3)	0.76135 (11)	0.0550 (5)	
H9A	0.4968	0.5679	0.7142	0.066*	
C10	0.68092 (17)	0.3624 (3)	0.84397 (12)	0.0565 (5)	
H10A	0.7504	0.2943	0.8656	0.068*	
C11	0.45564 (14)	0.6885 (2)	0.91621 (9)	0.0393 (4)	
H11A	0.3900	0.7203	0.9317	0.047*	
C12	0.56903 (15)	0.7736 (2)	0.96193 (9)	0.0382 (4)	
C13	0.65828 (16)	0.8061 (2)	0.92821 (10)	0.0458 (4)	
H13A	0.6477	0.7676	0.8745	0.055*	
C14	0.76122 (17)	0.8936 (2)	0.97250 (11)	0.0528 (5)	
H14A	0.8206	0.9166	0.9494	0.063*	
C15	0.77542 (17)	0.9468 (2)	1.05143 (12)	0.0529 (5)	
C16	0.69124 (17)	0.9189 (2)	1.08757 (11)	0.0509 (4)	
H16A	0.7039	0.9559	1.1419	0.061*	
C17	0.58720 (16)	0.8347 (2)	1.04174 (10)	0.0443 (4)	
H17A	0.5268	0.8179	1.0646	0.053*	

### Atomic displacement parameters (Å^2^)

**Table:**

	*U*^11^	*U*^22^	*U*^33^	*U*^12^	*U*^13^	*U*^23^
F1	0.0522 (7)	0.1184 (11)	0.1063 (10)	0.0076 (7)	0.0433 (7)	−0.0049 (9)
F2	0.0626 (8)	0.1346 (12)	0.0478 (6)	−0.0146 (7)	0.0117 (5)	0.0233 (7)
F3	0.0704 (8)	0.0765 (8)	0.0851 (9)	−0.0227 (7)	0.0113 (7)	−0.0198 (7)
O1	0.0622 (9)	0.1310 (14)	0.0718 (9)	−0.0220 (9)	0.0314 (8)	−0.0551 (10)
N1	0.0459 (8)	0.0450 (8)	0.0399 (7)	0.0012 (7)	0.0225 (6)	−0.0033 (6)
N2	0.0484 (9)	0.0524 (9)	0.0539 (9)	0.0084 (7)	0.0243 (7)	0.0022 (7)
N3	0.0605 (11)	0.0776 (12)	0.0593 (10)	−0.0020 (9)	0.0359 (9)	−0.0177 (9)
C1	0.0413 (11)	0.0612 (12)	0.0693 (13)	−0.0024 (9)	0.0276 (9)	−0.0047 (10)
C2	0.0383 (10)	0.0628 (12)	0.0636 (12)	−0.0012 (9)	0.0063 (9)	0.0031 (10)
C3	0.0467 (11)	0.0618 (11)	0.0412 (9)	−0.0114 (9)	0.0120 (8)	0.0041 (9)
C4	0.0416 (10)	0.0451 (9)	0.0442 (9)	−0.0038 (8)	0.0166 (8)	−0.0025 (8)
C5	0.0488 (10)	0.0527 (11)	0.0484 (10)	0.0081 (9)	0.0194 (8)	0.0069 (8)
C6	0.0578 (12)	0.0678 (13)	0.0562 (11)	0.0033 (10)	0.0312 (10)	0.0060 (10)
C7	0.0496 (10)	0.0573 (11)	0.0432 (9)	−0.0040 (9)	0.0212 (8)	−0.0076 (9)
C8	0.0428 (9)	0.0425 (9)	0.0371 (8)	0.0045 (7)	0.0202 (7)	0.0018 (7)
C9	0.0610 (12)	0.0677 (12)	0.0429 (10)	−0.0030 (10)	0.0264 (9)	−0.0078 (9)
C10	0.0490 (11)	0.0603 (12)	0.0664 (12)	0.0040 (9)	0.0279 (10)	−0.0105 (10)
C11	0.0416 (9)	0.0414 (9)	0.0387 (8)	0.0064 (7)	0.0188 (7)	0.0038 (7)
C12	0.0442 (9)	0.0348 (8)	0.0376 (8)	0.0078 (7)	0.0167 (7)	0.0023 (7)
C13	0.0543 (11)	0.0454 (10)	0.0409 (9)	−0.0011 (9)	0.0206 (8)	−0.0016 (8)
C14	0.0535 (11)	0.0493 (11)	0.0609 (11)	−0.0043 (9)	0.0266 (9)	0.0004 (9)
C15	0.0527 (11)	0.0401 (10)	0.0560 (11)	−0.0032 (9)	0.0065 (9)	−0.0048 (9)
C16	0.0632 (12)	0.0448 (10)	0.0422 (9)	0.0062 (9)	0.0151 (9)	−0.0045 (8)
C17	0.0534 (11)	0.0402 (9)	0.0432 (9)	0.0070 (8)	0.0218 (8)	−0.0009 (8)

### Geometric parameters (Å, º)

**Table:**

F1—C1	1.337 (2)	C5—H5A	0.9300
F2—C3	1.3382 (19)	C6—H6A	0.9300
F3—C15	1.341 (2)	C7—C8	1.465 (2)
O1—C7	1.199 (2)	C8—C11	1.323 (2)
N1—C9	1.323 (2)	C9—H9A	0.9300
N1—N2	1.351 (2)	C10—H10A	0.9300
N1—C8	1.414 (2)	C11—C12	1.443 (2)
N2—C10	1.298 (2)	C11—H11A	0.9300
N3—C9	1.300 (2)	C12—C13	1.383 (2)
N3—C10	1.336 (2)	C12—C17	1.385 (2)
C1—C6	1.345 (3)	C13—C14	1.359 (3)
C1—C2	1.355 (3)	C13—H13A	0.9300
C2—C3	1.357 (3)	C14—C15	1.361 (2)
C2—H2A	0.9300	C14—H14A	0.9300
C3—C4	1.365 (2)	C15—C16	1.352 (3)
C4—C5	1.373 (2)	C16—C17	1.362 (3)
C4—C7	1.479 (2)	C16—H16A	0.9300
C5—C6	1.362 (2)	C17—H17A	0.9300
			
C9—N1—N2	109.34 (14)	N1—C8—C7	113.87 (14)
C9—N1—C8	130.00 (15)	N3—C9—N1	110.95 (18)
N2—N1—C8	120.66 (12)	N3—C9—H9A	124.5
C10—N2—N1	101.56 (14)	N1—C9—H9A	124.5
C9—N3—C10	102.01 (15)	N2—C10—N3	116.13 (18)
F1—C1—C6	118.75 (17)	N2—C10—H10A	121.9
F1—C1—C2	117.86 (18)	N3—C10—H10A	121.9
C6—C1—C2	123.38 (17)	C8—C11—C12	129.55 (15)
C1—C2—C3	116.50 (18)	C8—C11—H11A	115.2
C1—C2—H2A	121.7	C12—C11—H11A	115.2
C3—C2—H2A	121.7	C13—C12—C17	117.96 (16)
F2—C3—C2	117.55 (17)	C13—C12—C11	123.14 (14)
F2—C3—C4	119.07 (16)	C17—C12—C11	118.80 (14)
C2—C3—C4	123.36 (16)	C14—C13—C12	120.92 (16)
C3—C4—C5	117.12 (16)	C14—C13—H13A	119.5
C3—C4—C7	121.05 (15)	C12—C13—H13A	119.5
C5—C4—C7	121.54 (16)	C13—C14—C15	118.50 (17)
C6—C5—C4	121.25 (17)	C13—C14—H14A	120.8
C6—C5—H5A	119.4	C15—C14—H14A	120.8
C4—C5—H5A	119.4	F3—C15—C16	118.53 (17)
C1—C6—C5	118.37 (17)	F3—C15—C14	118.29 (18)
C1—C6—H6A	120.8	C16—C15—C14	123.18 (17)
C5—C6—H6A	120.8	C15—C16—C17	117.69 (16)
O1—C7—C8	119.94 (16)	C15—C16—H16A	121.2
O1—C7—C4	119.85 (17)	C17—C16—H16A	121.2
C8—C7—C4	120.21 (15)	C16—C17—C12	121.70 (16)
C11—C8—N1	120.81 (15)	C16—C17—H17A	119.2
C11—C8—C7	125.23 (15)	C12—C17—H17A	119.2
			
C9—N1—N2—C10	0.01 (19)	O1—C7—C8—C11	166.70 (18)
C8—N1—N2—C10	−179.18 (15)	C4—C7—C8—C11	−12.5 (3)
F1—C1—C2—C3	−179.89 (17)	O1—C7—C8—N1	−9.9 (3)
C6—C1—C2—C3	1.0 (3)	C4—C7—C8—N1	170.88 (14)
C1—C2—C3—F2	−179.47 (17)	C10—N3—C9—N1	0.6 (2)
C1—C2—C3—C4	−1.0 (3)	N2—N1—C9—N3	−0.4 (2)
F2—C3—C4—C5	178.52 (16)	C8—N1—C9—N3	178.68 (16)
C2—C3—C4—C5	0.1 (3)	N1—N2—C10—N3	0.4 (2)
F2—C3—C4—C7	−7.6 (3)	C9—N3—C10—N2	−0.6 (2)
C2—C3—C4—C7	173.93 (18)	N1—C8—C11—C12	7.1 (3)
C3—C4—C5—C6	0.9 (3)	C7—C8—C11—C12	−169.35 (16)
C7—C4—C5—C6	−172.91 (17)	C8—C11—C12—C13	29.3 (3)
F1—C1—C6—C5	−179.18 (17)	C8—C11—C12—C17	−154.43 (17)
C2—C1—C6—C5	−0.1 (3)	C17—C12—C13—C14	0.8 (2)
C4—C5—C6—C1	−0.9 (3)	C11—C12—C13—C14	177.03 (16)
C3—C4—C7—O1	−46.8 (3)	C12—C13—C14—C15	0.8 (3)
C5—C4—C7—O1	126.8 (2)	C13—C14—C15—F3	178.87 (16)
C3—C4—C7—C8	132.36 (19)	C13—C14—C15—C16	−0.9 (3)
C5—C4—C7—C8	−54.0 (2)	F3—C15—C16—C17	179.59 (15)
C9—N1—C8—C11	−116.2 (2)	C14—C15—C16—C17	−0.6 (3)
N2—N1—C8—C11	62.8 (2)	C15—C16—C17—C12	2.3 (3)
C9—N1—C8—C7	60.6 (2)	C13—C12—C17—C16	−2.4 (2)
N2—N1—C8—C7	−120.40 (17)	C11—C12—C17—C16	−178.81 (15)
